# Validation of the wrist blood pressure measuring device Omron RS6 (HEM-6221-E) among obese Sudanese patients according to the European Society of Hypertension International Protocol Revision 2010

**DOI:** 10.12688/f1000research.26442.1

**Published:** 2020-10-30

**Authors:** Elrazi A. Ali, Saeed M. Omar, Yassin Ibrahim, Osama Al-Wutayd, Ishag Adam

**Affiliations:** 1 Faculty of Medicine, University of Khartoum, Khartoum, Sudan; 2 Faculty of Medicine, Gadarif University, Gadarif, Sudan, 249, Sudan; 3 Faculty of Medicine, University of Tabuk, Tabuk, P.O. Box 741, Saudi Arabia; 4 Department of Family and Community Medicine, Unaizah College of Medicine and Medical Sciences, Qassim University, Unaizah, Saudi Arabia; 5 Department of Obstetrics and Gynecology, Unaizah College of Medicine and Medical Sciences, Qassim University, Unaizah, Saudi Arabia

**Keywords:** validity, Omron RS6®, obesity, wrist, blood pressure

## Abstract

**Background:** Electronic devices for measuring blood pressure (BP) need to go through independent clinical validation as recommended by different authorities, both in general and specific populations. The aim of this study was to assess the validity of the Omron RS6 (HEM-6221-E) wrist oscillometric devices in obese Sudanese patients.

**Methods:** Of 90 obese individuals invited for recruitment, 33 were included in the study, and had their BP at the level of the wrist measured using Omron RS6 and standard mercury sphygmomanometer. Two observations were made and the mean was taken. BP differences between the two methods for the 33 participants were classified into three categories (≤5, ≤10, and ≤15 mmHg), according to the European Society of Hypertension-International Protocol revision 2010 (ESH-IP2) criteria. This was then used to assess the validity of the tested Omron RS6 device.

**Results:** Participants had a mean age of 56.97 years (standard deviation (SD), 8.75; range, 36-79). Average systolic blood pressure (SBP) was 146.21 mmHg (SD, 23.07; range, 107-182), and average diastolic blood pressure (DBP) was 93.82 mmHg (SD, 16.06; range, 67-128). There was a good agreement between the two observations using the OMRON RS6 and the standard sphygmomanometer: −4 to + 3 mmHg for SBP and −4 to +4 mmHg for DBP, with the mean difference of 1.73±1.11 mmHg for SBP and 1.49±1.02 mmHg for DBP.

**Conclusion: **Thus, the Omron RS6 (HEM-6221-E) is a valid and suitable measure of BP according to ESH-IP2.

## Introduction

Hypertension is defined as systolic blood pressure (SBP) ≥140 mmHg and/or diastolic blood pressure (DBP) ≥90 mmHg ^1^. It is a global public health issue, and is recognized to be the major factor contributing to the burden of heart disease, stroke, kidney failure and premature mortality and disability all over the world ^2^. In spite of this burden, a simple preventive and non-invasive measure such as checking and monitoring blood pressure (BP) is expected to decrease the associated cardiovascular mortality with hypertension dramatically ^3^. Hypertension is mostly symptomless particularly in the early stages unless there are complications or other reasons to reveal it, which is why many people go undiagnosed. Moreover, those who are diagnosed may not have access to treatment and may not be able to successfully control their hypertension long term. In order to ensure that the disease is well-controlled, BP should be monitored regularly.

Accurate BP readings via a reliable device is equally important, since this will influence diagnosis and treatment. Generally, there are electronic, mercury and aneroid devices that are used to measure BP. The World Health Organization recommends the use of affordable and reliable electronic devices that have the option to select manual readings, because mercury is toxic and aneroid devices need calibrations and trained personnel for use ^1^. Therefore, these electronic devices must be of certain accuracy. As a result, many BP measuring devices have been validated to meet international standards for the use by the general population ^4, 5^. However, few studies for the validation of electronic devices in specific groups, such as the elderly, infants and obese individuals, have been done.

For overweight and obese individuals, BP measurement is more challenging. First, obesity itself plays a major role in elevating BP through increasing insulin resistance and creating a cycle that eventually ends to metabolic syndrome ^6^. Secondly, obesity affects individuals in very different ways, and many obese people have an significantly increased arm circumference. Hence, if BP is not measured with an appropriately sized cuff it will lead to false high readings, which may conduce unnecessary treatment ^7^. In order to solve the problem of adiposity, some studies use the forearm instead of the arm to measure BP in obese individuals, which results in overestimated BP by approximately 7-15 mmHg – this has to be corrected with equations ^8^. Recently, a study targeting obese individuals compared the BP readings using two devices, one at the level of the arm (brachial artery) and the other at the wrist level (radial artery) to a standard method. These authors found that the electronic device's readings varied significantly from the readings obtained by the standard sphygmomanometer and did not meet the required criteria for obese adults ^5^. In this study, we aimed to evaluate BP measurements among obese hypertensive Sudanese patients using the radial artery (wrist level) compared to the standard brachial measured using a mercury sphygmomanometer and non-electronic stethoscope to attain more valuable information about the validity of using such a method.

## Methods

### Test device

Omron RS6 (HEM-6221-E) is a fully automated device for self/home BP measurement at the wrist level using the oscillometric method. Inﬂation is by automated fuzzy logic–controlled electric pump and deﬂation is by automatic pressure release valve. It measures 87 mm × 64 mm × 14 mm (width × height × depth). It has a single wrist cuff, which can be used for wrist size between 13.5–21.5 cm. The device demonstrates the pulse and BP (SBP and DBP) in a digital liquid crystal screen. The device can detect a BP range from 0 to 299 mmHg and pulse rate from 40 to 180 beats/min. Additionally, it can save the last 90 BP readings and it has a position sensor for the wrist and the ability to detect irregular pulse and body motion.

### Study details

A cross-sectional study was conducted at Gadarif Hospital outpatient clinic in eastern Sudan. Patients who fulfilled the following inclusion criteria were enrolled in the present study: obese (body mass index (BMI) ≥30 kg/m ^2^), adult (age ≥25 years), male and female hypertensive patients, able to give informed consent. BMI was computed from measured weight and height using standard methods. Individuals with abnormal rhythm or uncertainly DBP were excluded.

### Population recruitment

This study was conducted among the obese Sudanese population. A total of 90 participants volunteered to take part in the study and met the inclusion criteria, with at least 30 men and 30 women. Participants were invited to take part in the study during their regular doctor’s appointment.

Participants were recruited to ensure a uniform distribution of test pressures across the BP range: 90–180 mmHg for SBP and 40–130 mmHg for DBP. The participants were divided into three groups as per their SBP and DBP readings: SBP – low (90–129), medium (130–160), and high (161–180); for DBP – low (40–79), medium (80–100) and high (101–130). Readings outside these ranges were included and were either categorized as low or high. In each of the three SBP and DBP ranges, a minimum of 10 to 12 patient was included according to the European Society of Hypertension-International Protocol revision 2010 (ESH-IP2) guidelines with at least 10 men and 10 women in the sample size ( Table 1). 

**Table 1.** Screening and recruitment details.

Screening and recruitment		Blood pressure of recruited participants (n=33)				
Total screened	90			mmHg	n	On Rx
Reasons for exclusion from study		SBP	Low	<90	0	
* Ranges completed*	0			90-129	11	
* Range adjustment*	36		Medium	130–160	11	
* Arrhythmias*	0		High	161–180	9	
* Device failure*	0			>180	2	
* Poor quality sounds*	0					
* Cuff size unavailable*	0	DBP	Low	<40	0	
* Observer disagreement*	18			40–79	11	
* Distribution*	0		Medium	80–100	10	
Other reasons *	3		High	101–130	12	
Total excluded	57			>130	0	
Total recruited	33					

*Had to leave before for personal reason before completing the measurements. SBP, systolic blood pressure; DBP, diastolic blood pressure

### Data collection

***Procedure*.** In the outpatient clinic, BP measurements were taken in a room with a comfortable temperature, and ambient noise kept to minimum to avoid disruption during auscultation. Patients who fulfilled the inclusion criteria were allowed to rest for 10 minutes sitting on a chair in upright position with their legs uncrossed and back supported, with complete exposure of the left arm and forearm (at the time of BP checking) so there were no intervening clothes between the cuff and the arm, which might reduce the blood flow. Participants were encouraged to avoid talking or using mobile phones. The arm circumference was checked, and the suitable cuff applied. All the measurements were obtained from the left arm at the heart level.

BP measurements were obtained first by the standard mercury sphygmomanometer then alternating with the test device, so that we obtained nine consecutive BP measurements from each participant: five measurements from the standard mercury sphygmomanometer and four measurements from the tested device.

***Blood pressure measurements*.** The manufacturer of the Omron RS6 (HEM-6221-E) was asked to provide a standard model, and the automated device was used after familiarization sessions in the outpatient clinic for one week, during which time the team performed 12 test measurements and accustomed themselves to the device and the study. No problems in this familiarization period were encountered. 

For the standard test, two stethoscopes and two mercury sphygmomanometers with different cuff sizes were carefully checked prior to the study.

The working team composed of three personnel, two observers and a supervisor, well trained in BP measurement using standard mercury sphygmomanometer and stethoscope with well-fitting earpieces. They were of good health, hearing, and sight and able to follow the menisci at eye level from 40 mmHg to 180 mmHg. The two observers measured BP using two mercury sphygmomanometer and these readings were used as a reference. Observers were blinded from each other’s readings and from the device readings. They took BP measurement simultaneously and record them to the nearest 2 mmHg. The supervisor checked the agreement of BP values between the two observers so that any readings that varied by 4 mmHg or more were repeated. The supervisor measured the BP using the test wrist device. Two observations were made.

Before measuring BP, the observer measured the arm circumference in order to use the appropriate size cuff for the standard mercury sphygmomanometer: small cuff for an arm circumference 17–21.9 cm, a regular cuff for an arm circumference of 22–31.9 cm, a large cuff for an arm circumference of 32–42 cm and for patients with arm circumference more than 42 cm an extra-large cuff is used.

### Data analysis

The ESH-IP2 ^9^ was used as a guide for data analysis ^9^. Accordingly, data was analyzed, presented and expressed to assess the ability of the device to pass or fail to pass the validation protocol requirements. SPSS (version 22) and Microsoft Excel software were used to perform all data analysis. Measurements obtained by the two observers for the standard device were compared and their average was taken, which was later compared with the test device SBP and DBP. The numbers of differences for the tested device and the observer using mercury sphygmomanometer within 5, 10, and 15mmHg, were calculated for both SBP and DBP then the average differences of the values with the standard deviation between the SBP and DBP of the mercury and the tested device were obtained. Finally, Bland–Altman plots were performed for both SBP and DBP to show the differences of device-observer versus average device and observer values for all the 99 pairs of comparisons.

### Ethics approval and consent to participate

Ethical approval was received from the Ethics Committee at the Faculty of Medicine, Gadarif University, Sudan (reference number: 2017/09). Written informed consent to participate was collected from each participant before taking part in the research.

## Results

### Study population

In total, 90 obese participants were screened for inclusion into this study, 57 were excluded (36 due to range adjustment, 18 due to observers' disagreement and 3 left before completing the sequence of measurements for personal reasons). Therefore, data related to 33 participants (12 men and 21 women) who fulfilled the requirements and the criteria of the ESH-IP protocol were analyzed.

Mean (±standard deviation) age of participants was 56.97±8.75 years (range, 36–79 years); mean wrist circumference was 22.58±3.25 (range, 13–28 cm); mean height was 163.52±13.54 cm (range, 116–180; mean weight of 104.45±11.45 kg (range, 88–136); and mean BMI of 38.74±8.26 (range, 31.48–81.75). The mean BP values for SBP was 146.21±23.07 mmHg (range, 107–182 mmHg) and for DBP was 93.82±16.06 mmHg (range, 67–128 mmHg). Respondents' characteristics and BP measurements are summarized in Table 2 and Table 3.

**Table 2.** Participant demographics (n=33).

Demographic	Value
Gender, n (%)	
*Male*	12 (36.36)
*Female*	21 (63.64)
Age (years)	
*Range*	36–79
*Mean (SD)*	56.97 (8.75)
Arm circumference (cm)	
*Range*	33–90
*Mean (SD)*	43.45 (9.03)
Cuff for test device, n (%)	
*Standard*	10 (31.25)
*Large*	22 (68.75)
Wrist circumference (cm)	
*Range*	13–28
*Mean (SD)*	22.58 (3.25)
Height (cm)	
*Range*	116–180
*Mean (SD)*	163.52 (13.54)
Weight (kg)	
*Range*	88 – 136
*Mean (SD)*	104.45 (11.45)
BMI	
*Range*	31.48 – 81.75
*Mean (SD)*	38.74 (8.26)
Systolic blood pressure (mm Hg)	
*Range*	107–182
*Mean (SD)*	146.21 (23.07)
Diastolic blood pressure (mm Hg)	
*Range*	67–128
*Mean (SD)*	93.82 (16.06)

**Table 3.** Validation results.

Part 1					
	≤5mmHg	≤10mmHg	≤15mmHg	Grade 1	Mean±SD (mmHg)
**Pass requirement**					
Two of three	73	87	96		
All of	65	81	93		
**Achieved**					
SBP	97	99	99	Pass	1.91±1.47
DBP	98	99	99	Pass	1.78±1.47
Part 2					
	2/3 5mmHg		0/3 5mmHg	Grade 2	Grade 3
**Pass requirement**	≥24 participants		≤3 participants		
**Achieved**					
SBP	N=33		N=0	Pass	Pass
DBP	N=33		N=0	Pass	Pass
**Overall result: PASS**					

SBP, systolic blood pressure; DBP, diastolic blood pressure

**Figure 1.**
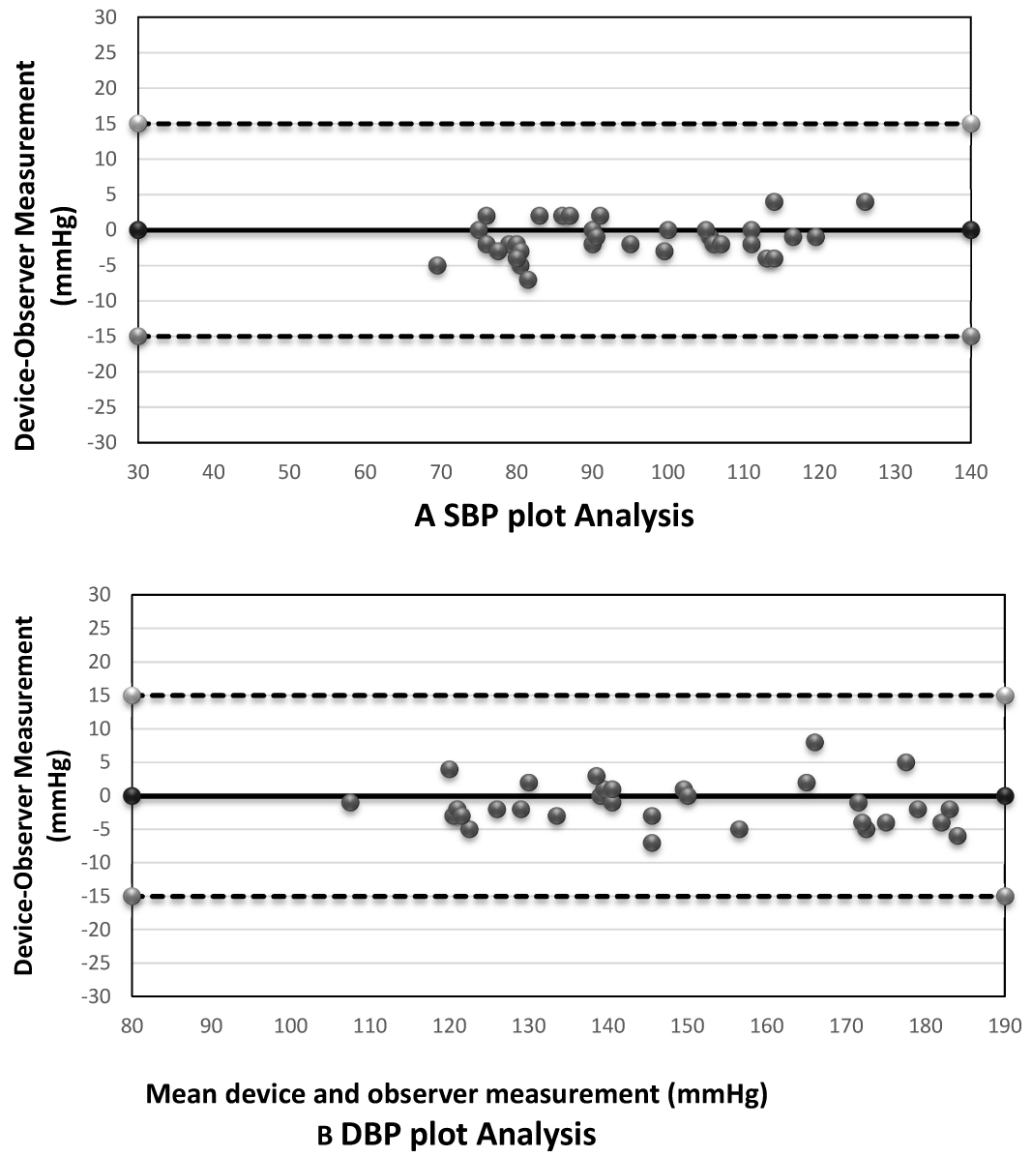
Bland–Altman plots of the differences between the BP measurements. ( **A**) Systolic blood pressure; ( **B**) diastolic blood pressure.

There was adequate agreement between the tested wrist BP measuring device (Omron RS6 (HEM-6221-E)) and the mercury sphygmomanometer ( Table 4). All the dots for both SBP and DBP measurements were inside the ±15 mmHg limits ( Figure 1, Table 5).

**Table 4.** Observations (n) in each SBP and DBP recruitment range.

SBP		DBP	
Overall range	111–185 mmHg	Overall range	66–130 mmHg
Low (<130)	26	Low (<80)	33
Medium (130 – 160)	40	Medium (80 – 100)	33
High (>160)	33	High (>100)	33
Maximum difference	14	Maximum difference	0.0

SBP, systolic blood pressure; DBP, diastolic blood pressure

**Table 5.** Observer differences.

	SBP (mmHg)	DBP (mmHg)	Repeated measurements
Observer 2 – Observer 1			
Range	-4 to +3	-4 to 4	
Mean difference (SD)	1.73 (1.11)	1.49 (1.02)	0.0

SBP, systolic blood pressure; DBP, diastolic blood pressure

## Discussion

In this study, the Bland-Altman plot showing the observers differences in SBP and SBP reveal that there is adequate agreement between the tested wrist BP measuring device (Omron RS6 (HEM-6221-E)) and the mercury sphygmomanometer ( Figure 1). The SBP and DBP plot reflects the overall distribution of measurements among study subjects. All the dots for both systolic and diastolic blood pressure were inside the ±15 mmHg limits.

A very recent study ^10^ also showed that the OMRON RS7 device fulfils the validation criteria of ESH-IP validation protocol in two independent study centres among subjects, showing inter-centre reproducibility.

In our study, the screening process to include and exclude participants was time consuming. In addition, there was a difficulty in recruiting participants with high blood pressure. According to the validation protocol of ESH-IP, three studies have already been carried out to confirm the validity of the Omron RS6 (HEM-6221-E) device. Validation among the general population confirmed the general validity of the device ^11^, however another study reported that the device failed to fulfil the ESH-IP revision 2010 requirements among obese subjects ^5^. Deutsch *et al.* used the Omron RS6 position sensor with PSON or PSOFF, which showed that the position sensor is important for the function of the device at the wrist level and it improves the accuracy of the measurements by decreasing variations in wrist height ^12^.

### Limitations

This study included only participants meaning that extrapolation of the results should be done with caution to individual who are not obese. Moreover, device accuracy needs to be adjusted to wrist circumference and position during measurement.

## Conclusion

The tested device, Omron RS6 (HEM-6221-E), achieved all the required standards for self/home measurement of blood pressure at the wrist level set by the ESH-IP, and accordingly would safely be recommended for personal use at home among obese patients provided that the manufacturer’s instructions are followed.

## Data availability

### Underlying data

Open Science Framework: Validation of the wrist blood pressure measuring device Omron RS6 (HEM-6221-E) among obese Sudanese patients compared with a standard mercury sphygmomanometer: a cross-sectional study according to the European Society of Hypertension International Protocol Revision 2010, https://doi.org/10.17605/OSF.IO/7S5D3 ^13^ (registered on 18 ^th^ October 2020: osf.io/w7dtn).

Data are available under the terms of the Creative Commons Zero "No rights reserved" data waiver (CC0 1.0 Public domain dedication).
