# Supplementary material for: Validation of the wrist blood pressure measuring device Omron RS6 (HEM-6221-E) among obese Sudanese patients according to the European Society of Hypertension International Protocol Revision 2010
Source: F1000Res. 2020 Oct 30;9:1284. [Version 1] doi: 10.12688/f1000research.26442.1 (PMC8503788; doi:10.12688/f1000research.26442.1)
Supplement: Peer review history

# Peer review: Reviewer response for version 1

**DOI:** 10.5256/f1000research.29192.r95237

**Published:** 2021-10-08

**Authors:** Yoshio Iwashima

**Affiliations:** 1 Division of Hypertension and Nephrology, Dokkyo Medical University, Tochigi, Japan

This study investigated the validity of the Omron RS6 (HEM-6221-E) wrist oscillometric devices in the blood pressure monitoring for 33 Obese Sudanese patients. This manuscript is well written and obtained results confirming their hypothesis. However, there are several studies that blood pressure levels were different between the level of the arm and that of the wrist. It should be noted that, based on much evidence, blood pressure is recommended to be measured in the upper arms, using an appropriate cuff size for the arm circumference. In the case of home blood pressure monitoring for obese patients, blood pressure should be measured at the level of the arm (brachial artery) using a larger cuff.

Is the work clearly and accurately presented and does it cite the current literature?

Yes

If applicable, is the statistical analysis and its interpretation appropriate?

Yes

Are all the source data underlying the results available to ensure full reproducibility?

Yes

Is the study design appropriate and is the work technically sound?

Yes

Are the conclusions drawn adequately supported by the results?

Yes

Are sufficient details of methods and analysis provided to allow replication by others?

Yes

Reviewer Expertise:

NA

I confirm that I have read this submission and believe that I have an appropriate level of expertise to confirm that it is of an acceptable scientific standard.

# Peer review: Reviewer response for version 1

**DOI:** 10.5256/f1000research.29192.r87886

**Published:** 2021-07-12

**Authors:** James A Hodgkinson

**Affiliations:** 1 Institute of Applied Health Research, University of Birmingham, Edgbaston, Birmingham, UK

The article presents a successful validation of a blood pressure monitor in an obese population. It satisfies the methodological requirements for a validation study and is written clearly and succinctly.

 It is to be noted (as the authors do) that a validation study of the same monitor in an equivalent population has been previously conducted and found the monitor failed validation. This is perfectly reasonable in itself, but perhaps raises the question of why the authors felt another validation study was warranted, and if they have any insights into the reason for the difference in results. Arguably related to this, some of the discussion of previous studies (at least the positive result for the monitor in the general non-obese population) should be in the background.

Is the work clearly and accurately presented and does it cite the current literature?

Yes

If applicable, is the statistical analysis and its interpretation appropriate?

Yes

Are all the source data underlying the results available to ensure full reproducibility?

Yes

Is the study design appropriate and is the work technically sound?

Yes

Are the conclusions drawn adequately supported by the results?

Yes

Are sufficient details of methods and analysis provided to allow replication by others?

Yes

Reviewer Expertise:

Blood pressure monitoring

I confirm that I have read this submission and believe that I have an appropriate level of expertise to confirm that it is of an acceptable scientific standard.
